# A New Dietary Fiber Can Enhance Satiety and Reduce Postprandial Blood Glucose in Healthy Adults: A Randomized Cross-Over Trial

**DOI:** 10.3390/nu15214569

**Published:** 2023-10-27

**Authors:** Simou Wu, Wen Jia, Huimin He, Jun Yin, Huilin Xu, Chengyuan He, Qinqiu Zhang, Yue Peng, Ruyue Cheng

**Affiliations:** 1 Department of Nutrition and Food Hygiene, West China School of Public Health and West China Fourth Hospital, Sichuan University, Chengdu 610041, China; simonomon@163.com (S.W.); 15881869162@163.com (W.J.); 2 Recovery Plus USA, New York, NY 10019, USA; hehuimin@rplushealth.com (H.H.); yinjun@rplushealth.com (J.Y.); xuhuilin@rplushealth.com (H.X.); hechengyuan@rplushealth.com (C.H.); 3 Sichuan Key Laboratory of Fruit and Vegetable Postharvest Physiology, College of Food Science, Sichuan Agricultural University, Ya’an 625014, China; 2020318064@stu.sicau.edu.cn (Q.Z.); pengyue2@stu.sicau.edu.cn (Y.P.)

**Keywords:** RPG dietary fiber, satiety, blood glucose, GLP-1

## Abstract

Background: Dietary fiber plays a potential role in regulating energy intake and stabilizing postprandial blood glucose levels. Soluble dietary fiber has become an important entry point for nutritional research on the regulation of satiety. Methods: this was a double-blind, randomized cross-over trial enrolling 12 healthy subjects to compare the effects of RPG (R+PolyGly) dietary fiber products (bread, powder, and capsule) and pectin administered with a standard meal on satiety, blood glucose, and serum insulin level. Results: Adding 3.8% RPG dietary fiber to bread significantly increased the volume, water content, hardness, and chewiness of bread compared to 3.8% pectin bread and white bread and significantly improved the sensory quality of bread. RPG bread had better appetite suppression effects at some time points than the other two groups and the best postprandial blood glucose lowering effects among the three groups. Administration of RPG capsules containing 5.6 g of RPG dietary fiber with meals improved satiety and reduced hunger compared to 6 g of RPG powder and 6 g of pectin, which had the greatest effect on suppressing appetite and reducing prospective food consumption. The peak level of serum glucagon-like peptide-1 (GLP-1) in the RPG capsule group (578.17 ± 19.93 pg/mL) was significantly higher than that in other groups at 0 min and 30 min after eating. RPG powder had the best effect in reducing postprandial blood glucose and increasing serum insulin levels; the total area under the curve (AUC) of serum insulin with RPG powder was higher than other groups (5960 ± 252.46 μU min/mL). Conclusion: RPG dietary fiber products can improve the sensory properties of food, reduce postprandial blood glucose, and enhance satiety, especially in capsule and powder forms. Further research on the physiological effects of RPG dietary fiber is required to facilitate its use as a functional ingredient in food products.

## 1. Introduction

Satiety refers to the feeling of fullness and satisfaction that occurs in response to an eating event and ends when the signals of food processing and absorption subside, inducing a feeling of hunger [1]. Satiety is a complex state influenced by many factors in addition to food intake [2]. Gut peptide hormones such as GLP-1, cholecystokinin, and peptide YY (PYY) modulate medium-term satiety. These hormones are released during the passage of food through the gastrointestinal tract, and these hormones not only inhibit food intake but also play a role in processing food [3]. Prolonged satiety after ingestion is influenced by insulin, glucose, amino acid concentrations in the blood, and the oxidation of nutrients in the liver [4]. The brain integrates signals from all the processes involved in hedonic and self-regulatory control of appetite, as well as those related to satiety [5,6]. Type 2 diabetes mellitus is a growing epidemic, affecting millions of people worldwide [7]. The disease is characterized by the inability of the body to produce enough insulin or to use insulin effectively, leading to high levels of glucose in the blood [8]. Large prospective cohort studies have unequivocally indicated that a high intake of dietary fiber may help normalize blood glucose and reduce insulin resistance and the risk of developing type 2 diabetes mellitus [9,10]. Therefore, reformulation of foods to potentially produce foods that can both suppress appetite and lower blood glucose while also being enjoyable to eat can help influence behavioral change and promote healthier food choices.

Dietary fiber is closely linked to satiety. Consumption of soluble, viscous, gelatinous, and more fermentable fiber from fruits and certain vegetables has been shown to increase satiety and reduce hunger [9]. Several studies have demonstrated the satiety-enhancing effects of β-glucan, rye bran flour, whole grain rye, or a mixed high-fiber diet [11]. For example, consumption of 8 g of fenugreek fiber at breakfast was found to significantly increase satiety and reduce energy intake at lunchtime in eight obese patients [12]. “Vitamin World^®^ Vegan Meal” Formula of Feihe is a novel protein-enriched fiber dietary supplement containing potato protease inhibitor II (PI2). It was found to prevent weight gain, increase satiety, and reduce food intake in rats [13]. Compared to regular pork meatballs, adding rye bran and pea fiber to pork meatballs significantly improved subjective satiety in healthy men [14]. Large prospective cohort studies have consistently shown a significant association between high-fiber intakes (25 g/day for women and 38 g/day for men) and a 20–30% reduction in the risk of developing type 2 diabetes mellitus [9]. Dietary fiber intervention in women with gestational diabetes mellitus ameliorates hyperglycemia, improves perinatal outcomes, and reduces the incidence of impaired postnatal glucose tolerance [15]. In an experimental study, soluble fiber from banana peels was shown to reduce food intake, lower blood glucose, improve hepatopancreatic function, increase the abundance of intestinal flora, and improve the IRS/PI3K/AKT pathway in diabetic mice [16].

Many different types of fiber have been reported to provide satiety benefits and lower blood glucose levels; however, there is limited research on the mechanisms of these responses and the extent of their health benefits [17]. Glucagon-like peptide-1 (GLP-1) has garnered considerable attention in recent decades due to its wide range of metabolic actions. Of note, intestinal GLP-1 has been suggested to be an endogenous satiation signal [18]. However, studies on dietary fiber have not found good results in terms of increasing satiety, reducing hunger, and increasing GLP-1 secretion [19,20]. It is used as a nutritional fortifier in bakery products, and the mode of action of dietary fiber in the body is not fully understood [21,22].

In our previous studies, high sugar and high acid were identified as necessary conditions for high methoxyl pectin to form a gel, and this phenomenon was due to the formation of hydrogen bonds between pectin molecules and its hydrophobic force. In contrast, the gel formation mechanism of low-methoxyl pectin (LMP) involved calcium and other divalent metals. Calcium pectate (CaP), a mixture of LMP and low-valence ions like calcium, has better gelation properties than LMP alone due to the egg-box-like model [23]. In an in vitro study, the gel viscosity formed with CaP was significantly higher than that of pectin alone, and with increasing concentrations of calcium ions, the gel viscosity showed an initial increase followed by a decrease [24]. This prompted us to speculate whether it could be correlated with gastric emptying, blood glucose, and satiety in population studies.

Therefore, we conducted a double-blind, randomized cross-over trial to investigate the effect of RPG dietary fiber (a complex of LMP and calcium) products (bread, powder, and capsule) on human satiety and to explore the effect of RPG dietary fiber in lowering blood glucose and insulin response. Our findings may reveal the potential application of RPG dietary fiber products in the prevention and treatment of chronic diseases such as type 2 diabetes mellitus.

## 2. Materials and Methods

### 2.1. Subjects

Twelve healthy adults (five females and seven males; mean age: 27.4 ± 3.4 years; mean body mass index [BMI]: 21.5 ± 1.9 kg/m^2^) participated in this study conducted at the Recovery Plus Clinic (Chengdu, China). Subjects were recruited through advertisements on the West China Campus of Sichuan University and social media, after which they were screened according to the inclusion/exclusion criteria. The inclusion criteria were as follows: age range 18–40 year; BMI 18.5–24.9 kg/m^2^; no history of intake of medications known to affect glucose tolerance in the preceding three months (e.g., oral contraceptives, acetylsalicylic acid, and thyroxine); ability to tolerate fasting for at least 10 h; no gastrointestinal dysfunction. The exclusion criteria were as follows: history of diabetes or abnormal glucose tolerance; use of hypoglycemic drugs or insulin to treat diabetes or other diseases (including coronary heart disease, hypertension, hyperlipidemia, and hyperthyroidism); smokers; alcohol consumers; pregnant and lactating women; history of surgery or trauma in the preceding three months; users of steroids, enzyme inhibitors, or antipsychotic drugs; and history of allergy to pectin, milk, eggs, and gluten.

All subjects were provided with detailed instructions regarding the study procedures, lifestyle requirements during the study period, and instructions for completing the questionnaire. Written consent was obtained from all subjects prior to their enrollment. This study was approved by the Ethics Committee of West China Fourth Hospital/West China School of Public Health, Sichuan University (approval number: Gwll2022111). The trial protocol is registered in the China Clinical Trial Registry (registration number: ChiCTR2300068194).

### 2.2. RPG Dietary Fiber

RPG dietary fiber is a specially modified viscous dietary fiber complex. Its active ingredients are LMP and calcium salt, which provide Ca^2+^. The RPG dietary fiber processing includes weighing the required ingredients (LMP and calcium salt), mixing them using a mixer, and sieving to obtain the product.

### 2.3. Study Design

This was a randomized, double-blind, cross-over trial. The experiment was divided into two phases. Phase I was a study of RPG dietary fiber as an ingredient in pasta-based bakery products. Phase II was a study of RPG dietary fiber in the form of capsules and powders taken with meals. In each phase, all subjects received one of the test meals according to a random sequence automatically generated using a random number table. All subjects were required to eat all of the meals in each experiment phase. All 12 subjects participated in both phases of the experiment and took part in all the sample trials. The interval between the two phases of the experiment was 14 days, with a gap of 3 days between each sample experiment within each phase.

The subjects were required to maintain the following dietary habits and lifestyle for 3 d before the start of the experimental day and during the washout period: a reasonable diet, regular meals, no overeating, no excessive dietary control, no alcohol consumption, regular work and rest, no late nights, no strenuous exercise on the day before the test, and no food or water after 10 p.m. on the night before the test. Subjects were followed up on and instructed regarding their diet during the study period using a dedicated recorder. All twelve subjects arrived at the Recovery Plus Clinic on the experimental day at 8:20 a.m. after 10–14 h of fasting using a convenient mode of transport. After resting for 10 min, all subjects consumed the designated control meal or the experimental meal at the stipulated time between 8:30 a.m. and 9:00 a.m.

#### 2.3.1. Study of RPG Dietary Fiber for Bread Application

In the study of RPG dietary fiber for bread application, there were three groups: the blank control group (white bread with no extra dietary fiber, B_C), the standard control group (3.8% pectin-added bread, B_Int0), and the RPG experimental group (3.8% RPG dietary fiber-added bread, B_RPG). Prior to conducting the main experiment, we pre-baked all samples, both with and without the addition of RPG and pure pectin, to determine whether there would be any significant alterations in the appearance and texture of white bread. And to eliminate potential bias and interference from the researchers, we ensured that the individuals responsible for producing the white bread were different from those responsible for distributing it to the study participants. This separation of roles was an essential aspect of maintaining the integrity of our research.

Blood glucose level was measured using the silicon-based continuous glucose detection system (CGM) (20213070871) at 10 min before meals and 15, 30, 45, 60, 90, and 120 min after eating.

Subjects were required to fill out the Appetite Assessment Questionnaires at the following time points: 5 min before the meal; every 15 min for 1 h after the meal; and every 30 min thereafter till 3.5 h after the meal. All meals were required to be eaten within 5–10 min from start to finish. For 3.5 h after the meal, no other food, water, or food-related temptations were allowed. The participants were allowed to perform simple daily activities, mainly in the sitting position.

#### 2.3.2. Study of RPG Dietary Fiber as Meal Preparations

Subjects were divided into four groups: blank control group (no extra supplements, C), standard control group (6 g pectin powder, Int0), RPG powder experimental group (6 g RPG powder, Powder), and RPG capsule experimental group (10 pieces of capsule with 5.6 g RPG in total, Cap). Ten minutes before the start of the meal on the experimental day, an antecubital vein needle (Jiangxi Huali Medical Equipment Co., Ltd., Xiajiang, China, YSGP/22G × 0.8 mm) was placed by a licensed nurse to collect a pre-meal venous blood sample by directly connecting the needle to the blood collection tube. After the meal, blood samples were taken every 30 min for up to 3.5 h using a disposable venous needle (Zhejiang Kandelai Medical Instrument Co., Ltd., Hangzhou, China, 21G 0.8 × 28 mm) connected to a blood collection tube. The blood samples collected were used to detect blood glucose, serum insulin, and the serum glucagon-like peptide-1 (GLP-1) index that reflects satiety. 

All twelve subjects completed the Appetite Assessment Questionnaires at different time points, as mentioned in Section 2.3.1. To minimize subjective bias, all series of the same type of meal in both phases were randomly assigned to different trial days. For example, the bread control meal and the RPG bread test meal were pre-randomized for all subjects on all trial days.

### 2.4. RPG Dietary Fiber Product Preparation

#### 2.4.1. Series Meals of RPG Bread Study

The bread was prepared at the Recovery Plus Nutritional Science Laboratory. The steps of preparation included the weighing of the dry and wet ingredients for later use. RPG dietary fiber or pectin was added to the dry ingredients; after mixing all the ingredients, the dough was kneaded. After the dough underwent the processes of relaxation, separation, secondary relaxation, shaping, and proofing, it was baked in the oven at 180 °C for 18 min and then cooled down to obtain the test bread.

Textural analysis of each dough and bread sample was carried out using a TA.XT plus Texture Analyzer (Stable Micro Systems, Godalming, UK) according to a previously reported method with minor modifications [25]. Briefly, each dough slice (of the same size) was tested on the Texture Analyzer using the P/36R test probe. The test mode was as follows: test speed 1.0 mm/s; compression distance 50%; two compression cycles of 5 s.

Moisture content was analyzed following the ICC standard method ICC 110/1 (ICC, 1994). The specific volume (mL/g) of each bread sample was measured and calculated using a previously reported method [26]. Sensory evaluation of each bread sample was carried out according to a previously described method with some modifications via a trained panel comprising ten participants (five males and five females) in triplicate [27,28]. Randomized bread samples were tested by each participant at the same time. The sensorial traits evaluated were texture, color, taste, texture, form, and acceptability. The scoring scale ranged from 0 to 15 or 0 to 20 (dislike to extreme like).

The bread used in B_C, B_Int0, and B_RPG was analyzed for nutrients, sensory evaluation, moisture content, and TPA texture analysis. The meals set up in each group were required to start at the specified time. The information on the meals in each group is shown in Supplementary Table S1.

#### 2.4.2. Series Meals of Study of RPG Dietary Fiber as Meal Preparations

The meals were prepared at the Recovery Plus Nutritional Science Laboratory. The steps for preparing the RPG powder included weighing RPG dietary fiber and other ingredients and mixing them with a three-dimensional mixer to obtain the final product. As for RPG capsules, 0.56 g of RPG dietary fiber was filled into a No. 0 gelatin capsule shell to obtain RPG capsules.

In all groups, participants were required to start eating meals at the prescribed time and complete food and beverage intake in 10–15 min. To minimize subjective bias, the paired beverages did not differ significantly from each other in terms of traits (viscosity), taste, and flavor. The information about the meals in each group is shown in Supplementary Table S2.

### 2.5. Subjective Appetite Evaluation

In the two-phase study, subjective appetite and satiety were assessed using the visual analog scale [29]. Specific indicators included eating desire, satiety, hunger, fullness, and prospective food consumption. Each indicator was evaluated on a scale of 0 to 100 points, indicating two extremes. For example, for the evaluation of hunger, 0 indicates “I am not hungry at all,” and 100 points indicate “I have never been hungrier.” The first questionnaire (t0) was filled out within 5 min before the subjects started to eat, followed by every 15 min from the first bite up to one hour and every 30 min thereafter up to 3.5 h; the time points were designated as t0, t15, t30, t45, t60, t90, t120, t150, t180, and t210. Overall Appetite Suppression Score (OASS) = [Satiety score + Fullness score + (100 − Hunger score) + (100 − Prospective food consumption score)] [30,31].

### 2.6. Blood Glucose and Serum Insulin

Serum was separated after centrifugation of the venous blood samples and frozen at 20 °C before further processing. Blood glucose levels were measured using the glucose oxidase method, and serum insulin levels were measured using the chemical radiation method. The correlation between fasting blood glucose and postprandial blood glucose responses was calculated using the trapezoid rule as total area under the curve (AUC) and incremental area under the curve (iAUC) [32].

### 2.7. Glucagon-like Peptide 1

In the RPG meal preparation series, blood samples were collected from the antecubital vein and centrifuged to separate the serum, which was frozen and stored at −20 °C. GLP-1 was detected using an enzyme-linked immunosorbent assay (Shanghai Jianglai Biotechnology Co., Ltd., Shanghai, China, Article No.: JL12839).

### 2.8. Statistical Analysis

Results are expressed as mean ± standard deviation. Statistical analyses were performed using SAS (version 9.3). The level of significance was set at *p* < 0.05. Based on previous studies, the results of each score related to subjective appetite and the results of OASS were analyzed and evaluated. Normally distributed quantitative variables were analyzed using the Shapiro–Wilk test.

## 3. Results

### 3.1. RPG Dietary Fiber for Bread Application

#### 3.1.1. Bread-Related Parameters

**Figure 1:**
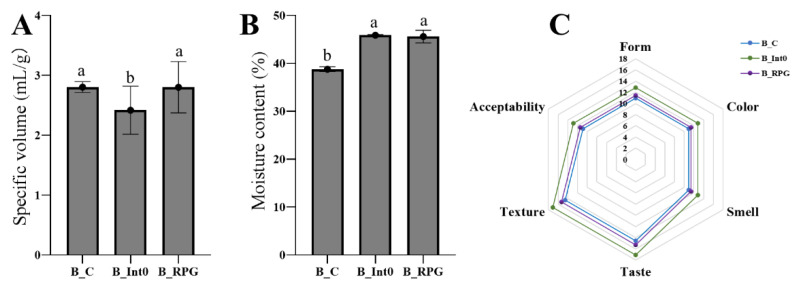
Specific volume of three groups of bread (**A**), moisture content (**B**), and sensory characteristics (**C**). “a, b” indicate significant differences between the two sets of data (*n* = 10).

As shown in Figure 1A, there was no significant difference in the specific volume of bread between the B_C and B_RPG groups (*p* > 0.05); the specific volume of bread in the B_Int0 group (mean: 2.42 ± 0.40 mL/g) was significantly lower than that in the B_C and B_RPG groups (*p* < 0.05). Moreover, there was no significant difference in the moisture content of the bread between the B_Int0 and B_RPG groups (*p* > 0.05), and both groups showed more than 45% moisture content (Figure 1B). However, the moisture content of the bread in the B_C group was significantly lower than that in the B_Int0 and B_RPG groups (*p* < 0.05). As seen in Figure 1C, the sensory scores of the breads in B_Int0, B_RPG, and B_C were in descending order (*p* < 0.05). The bread in the three groups had a smooth surface, normal volume, and good gloss, with a light-yellow color and a uniform internal color. Compared with the bread of group B_C, the bread of groups B_RPG and B_Int0 had a greater fermented aroma characteristic of bread; the bread of group B_Int0 was fluffier, had larger air holes on the cut surface, and had the highest texture score. The air holes on the cut surface of the bread of group B_RPG were relatively homogeneous.

Furthermore, as shown in Table 1, bread in the B_RPG group had the best hardness (483.20 ± 61.79 g), adhesiveness (280.05 ± 46.92), and chewiness (307.47 ± 31.66), significantly higher than the other two groups (*p* < 0.05). Bread in the B-Int0 group had the best springiness (0.941 ± 0.004 mm), resilience (0.35 ± 0.02), and cohesiveness (331.33 ± 34.79) (*p* < 0.05).

#### 3.1.2. Effect of RPG Dietary Fiber Bread on Subjective Appetite Evaluation

**Figure 2:**
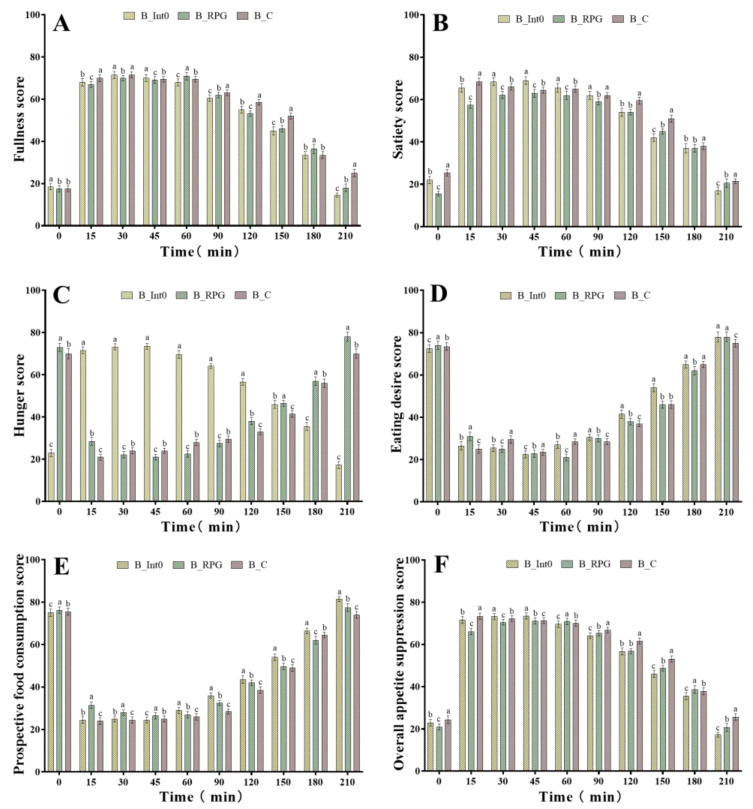
Fullness score of subjects consuming bread from groups B_Int0, B_RPG, and B_C (**A**), satiety score (**B**), hunger score (**C**), eating desire score (**D**), prospective food consumption score (**E**), and overall appetite suppression score (**F**). “a, b, c” indicate significant differences between the two sets of data (*n* = 12).

As shown in Figure 2A, after consumption of bread in the three groups, subjects in the B_RPG group had significantly higher fullness scores than those in the B_C and B_Int0 groups at 60 min and 180 min (*p* < 0.05). However, the highest fullness scores were found in the B_C group at 0 min and 45 min (*p* < 0.05), while the highest fullness scores were found in the B_Int0 group at 15 min, 90 min, 120 min, 150 min, and 210 min (*p* < 0.05). These results indicated that subjects who consumed the bread from the B_Int0 group had the greatest fullness. Moreover, the satiety scores of the B_RPG group were significantly lower than those of the B_Int0 group during the 0–210 min period of bread consumption and were also significantly lower than those of the B_C group during the 0–90 min period, but significantly higher than those of the B_C group at the 150 min, 180 min, and 210 min (*p* < 0.05) (Figure 2B). These findings indicated no significant satiety-enhancing effect of PRG dietary fiber bread. As shown in Figure 2C, at 30 min, 45 min, 60 min, and 90 min, the hunger scores of subjects in the B_RPG group were significantly lower than those of the B_Int0 and B_C groups (*p* < 0.05). At 0 min, 15 min, 120 min, and 150 min, the B_Int0 group had the lowest hunger scores. The B_C group had the lowest hunger scores at 180 min and 210 min (*p* < 0.05). These findings indicated a hunger suppression effect of RPG dietary fiber bread.

As shown in Figure 2D, subjects in the B_RPG group had the lowest eating desire scores at 30 min, 60 min, and 180 min (*p* < 0.05). However, subjects in the B_Int0 group had the lowest eating desire scores at 15 min, 90 min, 120 min, and 210 min. Moreover, as shown in Figure 2E, subjects in the B_RPG group had the lowest prospective food consumption scores at 180 min (*p* < 0.05). However, subjects in the B_Int0 group had the lowest prospective food consumption scores at 15 min, 30 min, 60 min, 90 min, 120 min, and 150 min (*p* < 0.05). As shown in Figure 2F, the overall appetite suppression scores of the B_RPG group were significantly higher than those of the B_Int0 and B_C groups at 60 min and 180 min (*p* < 0.05). However, at 0 min, 15 min, 90 min, 120 min, 150 min, and 210 min (*p* < 0.05), the overall appetite suppression scores of the B_Int0 group were significantly higher than the other two groups.

Thus, RPG dietary fiber bread showed some effect in enhancing satiety and suppressing hunger, but it was significantly less effective than bread made with equal proportions of pectin in enhancing fullness, reducing eating desire, and reducing food consumption. Moreover, it was also not as effective as pectin bread in suppressing overall appetite.

#### 3.1.3. Effect of RPG Dietary Fiber Bread on Postprandial Blood Glucose

**Figure 3:**
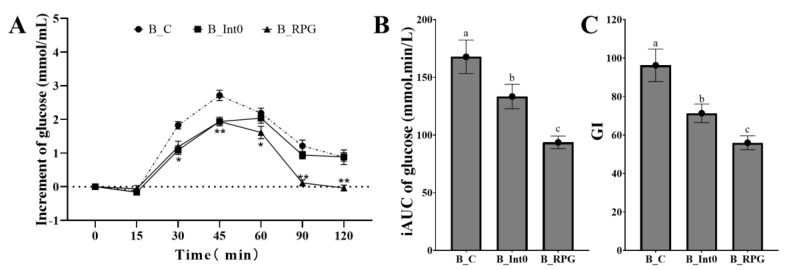
Increment of blood glucose of groups B_Int0, B_RPG, and B_C (**A**), iAUC of blood glucose (**B**), and GI (**C**) (*n* = 12). * indicates significant level of 0.05, and ** indicates a significant level of 0.01. “a, b, c” indicate significant differences between the two sets of data (*n* = 12).

As shown in Figure 3A, subjects in the B_RPG group had the lowest increment in blood glucose at 60 min, 90 min, and 120 min, significantly lower than the B_Int0 group and B_C group (*p* < 0.05). In the B_BPG group, the increment of blood glucose was even negative at 120 min after meals. Moreover, as shown in Figure 3B,C, the iAUC of blood glucose and GI value in the B_RPG group were also lower than the B_Int0 group and the B_C group (93.65 ± 5.48 mmol·min/L and 55.97 ± 3.62, respectively) (*p* < 0.05).

### 3.2. RPG Dietary Fiber as Meal Preparations

#### 3.2.1. Effect of RPG Dietary Fiber as Meal Preparations on Subjective Appetite Evaluation

**Figure 4:**
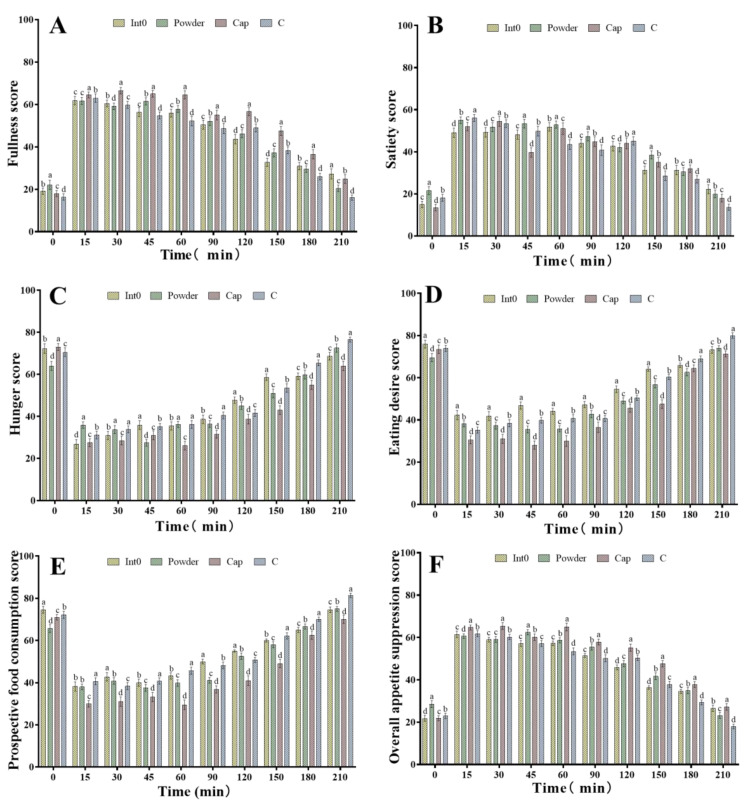
Fullness score of subjects consuming meals from groups Int0, Powder, Cap, and C (**A**), satiety score (**B**), hunger score (**C**), eating desire score (**D**), prospective food consumption score (**E**), and overall appetite suppression score (**F**). “a, b, c, d” indicate significant differences between the two sets of data (*n* = 12).

As shown in Figure 4A, all subjects in the Cap group demonstrated the highest fullness scores during the 15–180 min period, significantly higher than those in the Int0, C, and Powder groups (*p* < 0.05). Subjects in the Powder group demonstrated the highest satiety scores at 0 min, 45 min, 60 min, 90 min, and 150 min, which were significantly higher than those in the Int0 group, C group, and Cap groups (*p* < 0.05) (Figure 4B). In the Cap group, satiety scores were significantly higher than those in the other three groups at 30 min and 180 min (*p* < 0.05). Furthermore, as shown in Figure 4C, the subjects in the Cap group demonstrated the lowest hunger sensation scores from 60 min to 210 min, which were significantly lower than those in the Int0, C, and Powder groups (*p* < 0.05). Moreover, the Powder group also demonstrated significantly lower satiety scores than the other three groups at 0 min and 45 min (*p* < 0.05). RPG dietary fiber powder and capsule significantly improved subjects’ fullness or satiety and reduced post-meal hunger.

In addition, as shown in Figure 4D, subjects in the Cap group had the lowest eating desire scores from 15 min to 210 min after the meal, which were significantly lower than those in the Int0, C, and Powder groups (*p* < 0.05). The eating desire scores of the Powder group were also significantly lower than the other three groups at 0 min (*p* < 0.05).

Moreover, as shown in Figure 4E, subjects in the Cap group also had the lowest prospective food consumption scores from 15 min to 210 min after the meal, which were significantly lower than those in the Int0, C, and Powder groups (*p* < 0.05). The prospective food consumption scores of the Powder group were also significantly lower than those of the other three groups at 0 min (*p* < 0.05). As shown in Figure 4F, subjects in the Cap group also had the highest overall appetite suppression scores from 15 min to 210 min after the meal, which were significantly higher than those in the Int0, C, and Powder groups (*p* < 0.05). The overall appetite suppression scores of the Powder group were also significantly lower than those of the other three groups at 0 min (*p* < 0.05). Therefore, compared to powder and pectin, RPG dietary fiber capsules were found to have the best effect of suppressing eating desire and reducing prospective food consumption, and the overall appetite suppression effect was significantly better than that of pectin and powder.

**Figure 5:**
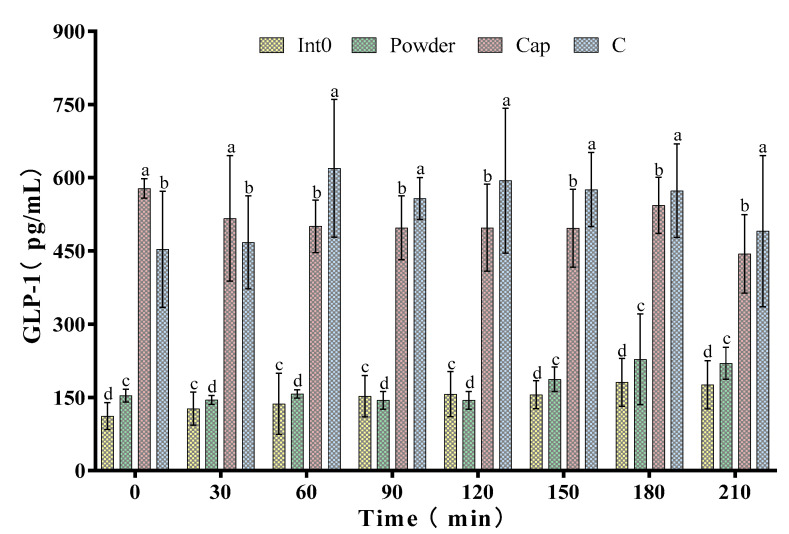
Glucagon-like peptide 1 (GLP-1) of subjects consuming meals from groups Int0, Powder, Cap, and C. “a, b, c, d” indicate significant differences between the two sets of data (*n* = 12).

As shown in Figure 5, the GLP-1 level in the Cap group was higher than the Int0, C, and Powder groups at 0 min and 30 min. At 0 min, GLP-1 concentration in the Cap group reached the peak value of 578.17 ± 19.93 pg/mL. Subsequently, GLP-1 concentration in the Cap group showed irregular changes but was significantly higher than that in the Int0 and Powder groups (*p* < 0.05). However, the GLP-1 concentration in the C group was higher than the Int0, Cap, and Powder groups at 60 min, 90 min, 120 min, 150 min, 180 min, and 210 min (*p* < 0.05). Especially 60 min after meal, GLP-1 concentration in the C group (619.17 ± 141.18 pg/mL) was 4.52 times that in the Int0 group and 3.94 times that in the Powder group.

#### 3.2.2. Effect of RPG Dietary Fiber as Meal Preparations on Postprandial Blood Glucose

**Figure 6:**
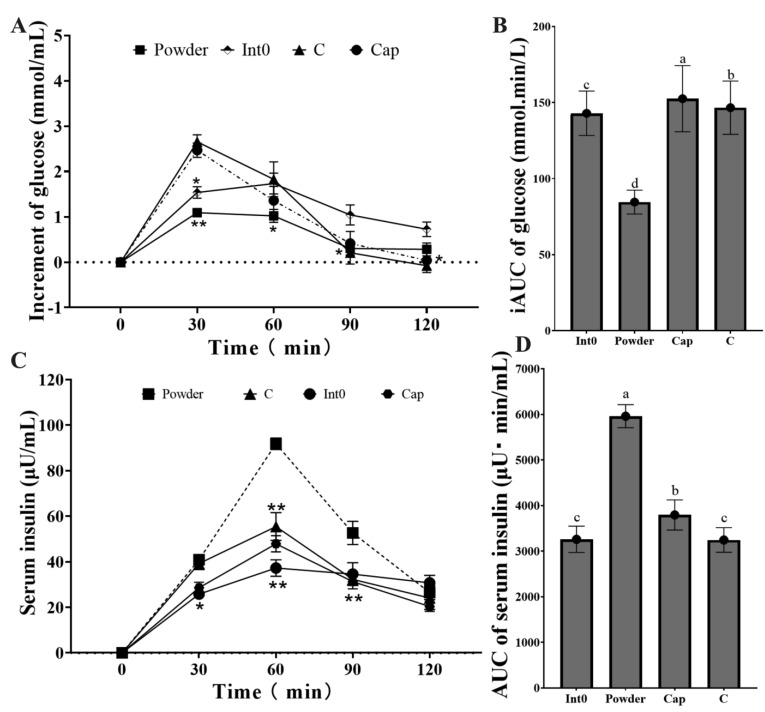
Increment of blood glucose from groups Int0, Powder, Cap, and C (**A**), iAUC of blood glucose (**B**), Serum insulin (**C**), and AUC of serum insulin (**D**). * indicates significant level of 0.05, and ** indicates a significant level of 0.01. “a, b, c, d” indicate significant differences between the two sets of data (*n* = 12).

The increment of blood glucose in the Powder group was lowest at 30 min and 60 min (*p* < 0.05) (Figure 6A). The iAUC of blood glucose in the Powder group (84.57 ± 7.82 mmol min/L) was significantly lower than that in the Int0, C, and Cap groups (*p* < 0.05) (Figure 6B). Moreover, the serum insulin level in the Powder group was highest at 60 min and 90 min, significantly higher than the other three groups (*p* < 0.05) (Figure 6C,D). The AUC of serum insulin in the Powder group (5960 ± 252.46 μU min/mL) was significantly higher than that in the Int0, C, and Cap groups (*p* < 0.05).

### 3.3. Side Effects

In this study, two subjects who consumed RPG capsules experienced mild flatulence, which recovered the next day. No gastrointestinal symptoms such as nausea, vomiting, abdominal distension, abdominal pain, or diarrhea were observed. None of the subjects experienced weight loss.

## 4. Discussion

### 4.1. Effect of RPG Dietary Fiber Bread on Human Satiety and Blood Glucose

As a new type of nutrient, the use of dietary fiber has gradually been extended from the fields of health foods to the field of dairy, beverages, baking, and other traditional foods [33]. Several studies have reported the good application value of soluble dietary fiber in bread. The addition of soluble dietary fiber to bread can not only increase the dietary fiber content, control postprandial blood sugar levels, and increase the nutritional value of bread, but also significantly improve the texture, appearance, and sensory properties of bread [34,35]. This can play a role in managing and preventing type 2 diabetes [36].

Soluble dietary fiber is rich in a variety of polysaccharide compounds that exhibit a certain degree of adhesion. The molecular structure of polysaccharides is similar to the three-dimensional network structure. With the addition of soluble dietary fiber to the flour, the combination of proteins in the flour forms a larger lattice structure, which can inhibit the aging of starch and play a role in improving the taste and texture of the flour [37,38,39].

In the present study, RPG dietary fiber bread showed better swelling ability, and the specific volume of bread was similar to that of ordinary white bread and significantly higher than that of pectin bread. In addition, the water content of both RPG fiber bread and pectin bread was more than 45%, which was significantly higher than that of white bread. This indicated that both RPG dietary fiber and pectin can increase the water retention capacity of bread, which is consistent with the findings of Almeida et al. [40,41]. The results of the sensory evaluation showed that pectin bread had the highest score, followed by RPG fiber bread. Both pectin bread and RPG fiber bread showed significantly better sensory attributes than white bread. Compared with white bread, RPG dietary fiber bread and pectin bread had a more characteristic fermentation aroma. Pectin bread was fluffier and had larger air holes in the cut surface, while the RPG dietary fiber bread had relatively more uniform air holes in the cut surface. This suggests that the addition of dietary fiber improves the leavening properties of bread [34]. Furthermore, RPG fiber bread had the best hardness, adhesiveness, and chewiness, while pectin bread had the best springiness, resilience, and cohesiveness. These results indicated that the addition of RPG fiber can increase hardness and improve the color, texture, and sensory attributes of bread.

Interestingly, human studies have found that RPG dietary fiber bread has a significantly better effect on enhancing satiety and suppressing hunger compared to white bread. However, in terms of increasing fullness, reducing eating desire, and reducing prospective food consumption, RPG dietary fiber bread was found to be significantly less effective than pectin bread. Moreover, RPG dietary fiber bread was not as effective as pectin bread in suppressing appetite. This may be attributed to the lower pectin content in RPG dietary fiber bread compared to pectin bread [42]. The bread preparation process is also a key determinant of the satiety of RPG dietary fiber bread, and, therefore, optimization of bread-related process parameters is also necessary [43,44].

Furthermore, compared to white bread, RPG dietary fiber bread significantly controlled the increment in blood glucose in subjects during the 30–150 min postprandial period. This may be attributable to the fact that RPG dietary fiber is more viscous than pectin, which slows gastric emptying and also reduces the rate of starch digestion and glucose absorption in the small intestine [45]. Moreover, subjects who consumed RPG dietary fiber bread showed minimal iAUC of blood glucose and GI value. The blood glucose results in this study were consistent with those reported by Brand-Miller et al. [46,47]. The addition of RPG dietary fiber to bread can play a role in lowering postprandial blood glucose levels in healthy people. RPG dietary fiber in bread is certainly an interesting application, which will broaden the application of the novel dietary fiber in the flour industry.

### 4.2. RPG Dietary Fiber as Meal Preparations on Human Satiety and Blood Glucose

In addition to being applied to bread, RPG dietary fiber can also be added to drinks to make drinks rich in dietary fiber. In addition, dietary fiber capsules are also the most commonly used dietary supplements [48]. In this study, RPG powder and capsule significantly improved fullness or satiety and reduced post-meal hunger. Compared to RPG dietary fiber powder and pectin, RPG dietary fiber capsules showed a better effect in suppressing eating desire and reducing prospective food consumption, and the overall appetite suppression effect was significantly better than that of pectin and RPG dietary fiber powder. RPG capsules were found to be the most effective application form of RPG dietary fiber. This is related to the slow release of dietary fiber capsules, which can provide a sustained sense of satiety for up to 3.5 h [49].

In the dairy industry, there has been a significant increase in the number of new high-fiber yogurts and milkshakes [50,51]. In snacks, the addition of fiber to chocolate bars and crisps has become commonplace [52,53]. Soluble dietary fibers such as chicory root fiber and oligofructose have prebiotic properties and improve the texture and oral feel of products [43,54]. Therefore, further efforts should be made for the application of PRG dietary fiber in other food fields. GLP-1 is a hormone produced by intestinal cells that increases insulin secretion, inhibits glucagon secretion, and slows gastric emptying, which is positively correlated with satiety [55,56]. However, in the present study, the RPG dietary fiber capsule group had significantly lower levels of GLP-1 than the blank control group during the 60–210 min period, which seems to be the exact opposite of the results for satiety. With an in-depth understanding of GLP-1′s satiety-inducing mechanisms, we found that intestinal GLP-1 secretion occurs during meals. In rodents and humans, GLP-1 concentrations in plasma peak at the earliest measurement time point after the start of feeding, which would explain the 0 min maximum GLP-1 concentration in RPG dietary fiber capsules [18]. Therefore, the experimental results are consistent with the findings of Pannacciulli and Williams et al. Postprandial GLP-1 response is positively associated with changes in neuronal activity of brain areas implicated in satiety and food intake regulation in humans [57,58]. GLP-1 results further verified the effect and mechanism of the RPG dietary fiber capsule on enhancing satiety.

Moreover, the increment of blood glucose after intake of RPG dietary fiber powder was the lowest, and the iAUC of blood glucose in the powder was also the lowest. Compared to RPG dietary fiber capsules, RPG dietary fiber powder significantly controlled the postprandial increase in blood glucose. This was likely attributable to the fact that RPG dietary fiber powder directly combines with gastric juices to form a higher viscosity fiber complex in the stomach, slowing gastric emptying and reducing the rate of glucose absorption in the small intestine [59,60]. Moreover, the serum insulin level was highest after the consumption of RPG dietary fiber powder, followed by RPG dietary fiber capsules. Insulin is associated with increased satiety, reduced blood glucose, and decreased hunger and energy intake in normal-weight subjects [61]. Furthermore, there is evidence that insulin is a regulator of ghrelin suppression [62,63]. This explains the increased satiety and decreased appetite observed after consumption of RPG dietary fiber powder and capsule.

## 5. Conclusions

In this study, the addition of RPG dietary fiber significantly increased the specific volume, moisture content, hardness, and chewiness of the bread and significantly improved its sensory attributes. RPG dietary fiber bread had limited effects on enhancing satiety and suppressing hunger compared to white bread, which was less effective than pectin bread in appetite suppression. However, compared to pectin bread and white bread, RPG dietary fiber bread had the best postprandial blood glucose-lowering effect. Our findings support the potential role of the consumption of RPG dietary fiber as a measure to prevent obesity and the development of type 2 diabetes. RPG dietary fiber powder and capsules significantly improved the feeling of satiety and fullness and reduced post-meal hunger in subjects compared to pectin. In particular, RPG dietary fiber capsules were found to be most effective in enhancing satiety, and the significantly higher serum GLP-1 level might be the underlying mechanism. In addition, compared to RPG dietary fiber capsules, RPG dietary fiber powder more directly controlled postprandial blood glucose and maintained serum insulin levels.

However, some limitations of this study should be considered while interpreting the results. The number of subjects in this study was limited because of the requirement for obtaining blood samples. Moreover, the study population exclusively comprised healthy adults, and our findings may not be entirely generalizable to patient populations. Considering these limitations, larger studies enrolling patients in addition to healthy subjects are required to verify the specific mechanism of the effect of RPG dietary fiber in the prevention and treatment of chronic diseases and further optimize the development of PRG dietary fiber products with better satiety and blood glucose control.

## Figures and Tables

**Table 1:** Effect of RPG dietary fiber on bread texture.

Groups	Hardness/g	Springiness/mm	Chewiness/mJ	Cohesiveness	Adhesiveness	Resilience
B_C	404.97 ± 41.56 ^b^	0.913 ± 0.04 ^b^	255.25 ± 41.76 ^b^	0.69 ± 0.11 ^b^	280.05 ± 46.92 ^b^	0.32 ± 0.06 ^c^
B_Int0	378.04 ± 25.37 ^c^	0.941 ± 0.004 ^a^	249.05 ± 11.88 ^c^	0.70 ± 0.02 ^a^	264.77 ± 12.77 ^c^	0.35 ± 0.02 ^a^
B_RPG	483.20 ± 61.79 ^a^	0.928 ± 0.01 ^a^	307.47 ± 31.66 ^a^	0.69 ± 0.02 ^c^	331.33 ± 34.79 ^a^	0.33 ± 0.02 ^b^

Letter marking: “^a^, ^b^, ^c^” indicate significant differences between the two sets of data (*n* = 10).

## Data Availability

The datasets used and/or analyzed during the current study are available from the corresponding author upon reasonable request.

## Supplementary Materials

The following supporting information can be downloaded at https://www.mdpi.com/article/10.3390/nu15214569/s1, Table S1: information of meals in each group; Table S2: information of meals of RPG dietary fiber as meal preparations.
