# Supplementary material for: A New Dietary Fiber Can Enhance Satiety and Reduce Postprandial Blood Glucose in Healthy Adults: A Randomized Cross-Over Trial
Source: Nutrients. 2023 Oct 27;15(21):4569. doi: 10.3390/nu15214569 (PMC10648557; doi:10.3390/nu15214569)
Supplement: Supplementary file 1 [file nutrients-15-04569-s001.zip › nutrients-2678609-supplementary.pdf]

Supplementary information

A new dietary fiber can enhance satiety and reduce postprandial blood glucose in healthy adults: a randomized cross-over trial

Simou Wu[1], Wen Jia[1], Huimin He[2], Jun Yin[2], Huilin Xu[2], Chengyuan He[2], Qinqiu Zhang[3], Yue Peng[3], Ruyue Cheng[1] *

[1] Department of Nutrition and Food Hygiene, West China School of Public Health and West China Fourth Hospital, Sichuan University, 610041, Chengdu, China

[2]Recovery Plus USA, New York, NY 10019, USA

[3] Sichuan Key Laboratory of Fruit and Vegetable Postharvest Physiology, College of Food Science, Sichuan Agricultural University, 625014, Ya'an, China

*Corresponding author: Ruyue Cheng

E-mail: ruyuecheng1993@163.com

Address: Department of Nutrition and Food Hygiene, West China School of Public Health and West China Fourth Hospital, Sichuan University, No.16, 3rd section, South Renmin Road, 610041 Chengdu, Sichuan, China

Table S1 Information of meals in each group

Groups	Food Information	Dietary fiber additions	Energy（kcal）	Carbohydrate energy ratio (%)	Protein energy ratio（%）	Fat energy ratio（%）	Dietary fiber Energy ratio (%)	Purified water with meals（mL）
B_C	White bread 141 g	0	341	58.6	15	22.8	3.7	250
B_Int0	Pectin bread 154 g	Pectin 3.8%	355	53.6	16.5	24.2	5.6	250
B_RPG	RPG dietary fiber bread 152 g	RPG dietary fiber 3.8%	353	53.9	16.5	24.0	5.6	250

Note: The bread for B_Int0 was supplemented with pectin, the main ingredient in the RPG dietary fiber complex, in the same amount as the RPG experimental meal, both 3.8%.

Table S2 Information of meals of RPG dietary fiber as meal preparations

Groups	Food information	Energy（kJ）	Carbohydrate (g)/energy ratio (%)	Protein (g)/energy ratio (%)	Fat (g)/energy ratio (%)	Dietary fiber (g)/energy ratio (%)
C	Commercially available bean paste bun 105 g	1193.9	53/75.5	5.78/8.2	5.3/16.3	0
	Tangerine drink 150 mL	66.3	1.8/46.2	0	0	0
Int0	Commercially available bean paste bun 105 g	1193.9	53/75.5	5.78/8.2	5.3/16.3	0
	Tangerine drink 150 mL（pectin dietary fiber powder 6 g）	66.3	1.8/46.2	0/0	0/0	4.2/53.8
Powder	Commercially available bean paste bun 105 g	1193.9	53/75.5	5.78/8.2	5.3/16.3	0
	Tangerine drink 150 mL（RPG dietary fiber powder 6 g）	66.3	1.8/46.2	0/0	0/0	4.2/53.8
Cap	Commercially available bean paste bun 105 g	1193.9	53/75.5	5.78/8.2	5.3/16.3	0
	Tangerine drink 150 mL + RPG capsule 10 piece（RPG dietary fiber 5.6 g）	50	1.21/41.5	0/0	0/0	3.64/58.5
